# Supplementary material for: Allosteric regulation and crystallographic fragment screening of SARS-CoV-2 NSP15 endoribonuclease
Source: Nucleic Acids Res. 2023 Apr 28;51(10):5255–70. doi: 10.1093/nar/gkad314 (PMC10250223; doi:10.1093/nar/gkad314)
Supplement: gkad314_Supplemental_Files [file gkad314_supplemental_files.zip › Supplementary datasets.zip!/224/Refine_9/01-BUSTER/Cycle-3/shell.01/R_Rfree.html]

# Rxpct factors in resolution bins at cycle 0


|  |  |  |  |  |  |  |  |  |
| --- | --- | --- | --- | --- | --- | --- | --- | --- |
|  | **<d> (Å)**  Rxpct\_Work | Rxpct\_Free | Rxpct\_All | |  |  |  |  |
|  | 15.0574 | 0.3628 | 0.5069 | 0.3678 |  |
|  | 8.6934 | 0.2372 | 0.2621 | 0.2387 |  |
|  | 6.7339 | 0.2359 | 0.2526 | 0.2369 |  |
|  | 5.6912 | 0.2259 | 0.2802 | 0.2276 |  |
|  | 5.0191 | 0.1834 | 0.2306 | 0.1863 |  |
|  | 4.5400 | 0.1692 | 0.1907 | 0.1703 |  |
|  | 4.1762 | 0.1764 | 0.1886 | 0.1771 |  |
|  | 3.8878 | 0.1820 | 0.2280 | 0.1840 |  |
|  | 3.6520 | 0.1869 | 0.2308 | 0.1890 |  |
|  | 3.4544 | 0.1835 | 0.2263 | 0.1860 |  |
|  | 3.2858 | 0.1924 | 0.2303 | 0.1945 |  |
|  | 3.1397 | 0.1935 | 0.2185 | 0.1946 |  |
|  | 3.0115 | 0.1925 | 0.2143 | 0.1934 |  |
|  | 2.8978 | 0.2002 | 0.1974 | 0.2000 |  |
|  | 2.7961 | 0.2006 | 0.2279 | 0.2019 |  |
|  | 2.7044 | 0.2067 | 0.2299 | 0.2079 |  |
|  | 2.6212 | 0.2071 | 0.2415 | 0.2091 |  |
|  | 2.5452 | 0.2191 | 0.2407 | 0.2203 |  |
|  | 2.4754 | 0.2243 | 0.2947 | 0.2284 |  |
|  | 2.4111 | 0.2427 | 0.2601 | 0.2435 |  |
|  | **Overall** | 0.2036 | 0.2350 | 0.2052 |  |

# Rxpct factors in resolution bins at cycle 1


|  |  |  |  |  |  |  |  |  |
| --- | --- | --- | --- | --- | --- | --- | --- | --- |
|  | **<d> (Å)**  Rxpct\_Work | Rxpct\_Free | Rxpct\_All | |  |  |  |  |
|  | 15.0574 | 0.3686 | 0.5146 | 0.3736 |  |
|  | 8.6934 | 0.2404 | 0.2632 | 0.2417 |  |
|  | 6.7339 | 0.2372 | 0.2534 | 0.2382 |  |
|  | 5.6912 | 0.2246 | 0.2824 | 0.2265 |  |
|  | 5.0191 | 0.1819 | 0.2314 | 0.1849 |  |
|  | 4.5400 | 0.1676 | 0.1901 | 0.1688 |  |
|  | 4.1762 | 0.1746 | 0.1912 | 0.1756 |  |
|  | 3.8878 | 0.1800 | 0.2261 | 0.1820 |  |
|  | 3.6520 | 0.1860 | 0.2320 | 0.1882 |  |
|  | 3.4544 | 0.1833 | 0.2263 | 0.1858 |  |
|  | 3.2858 | 0.1924 | 0.2319 | 0.1946 |  |
|  | 3.1397 | 0.1947 | 0.2186 | 0.1957 |  |
|  | 3.0115 | 0.1937 | 0.2155 | 0.1946 |  |
|  | 2.8978 | 0.2015 | 0.1986 | 0.2014 |  |
|  | 2.7961 | 0.2025 | 0.2283 | 0.2037 |  |
|  | 2.7044 | 0.2062 | 0.2301 | 0.2074 |  |
|  | 2.6212 | 0.2072 | 0.2402 | 0.2091 |  |
|  | 2.5452 | 0.2182 | 0.2439 | 0.2196 |  |
|  | 2.4754 | 0.2230 | 0.2939 | 0.2272 |  |
|  | 2.4111 | 0.2406 | 0.2579 | 0.2413 |  |
|  | **Overall** | 0.2035 | 0.2359 | 0.2052 |  |

# Rxpct factors in resolution bins at cycle 2


|  |  |  |  |  |  |  |  |  |
| --- | --- | --- | --- | --- | --- | --- | --- | --- |
|  | **<d> (Å)**  Rxpct\_Work | Rxpct\_Free | Rxpct\_All | |  |  |  |  |
|  | 15.0574 | 0.3643 | 0.5081 | 0.3693 |  |
|  | 8.6934 | 0.2371 | 0.2610 | 0.2385 |  |
|  | 6.7339 | 0.2335 | 0.2504 | 0.2345 |  |
|  | 5.6912 | 0.2213 | 0.2775 | 0.2231 |  |
|  | 5.0191 | 0.1797 | 0.2293 | 0.1827 |  |
|  | 4.5400 | 0.1668 | 0.1897 | 0.1680 |  |
|  | 4.1762 | 0.1734 | 0.1910 | 0.1744 |  |
|  | 3.8878 | 0.1798 | 0.2258 | 0.1819 |  |
|  | 3.6520 | 0.1858 | 0.2293 | 0.1879 |  |
|  | 3.4544 | 0.1828 | 0.2262 | 0.1853 |  |
|  | 3.2858 | 0.1910 | 0.2316 | 0.1933 |  |
|  | 3.1397 | 0.1930 | 0.2157 | 0.1940 |  |
|  | 3.0115 | 0.1925 | 0.2123 | 0.1933 |  |
|  | 2.8978 | 0.2006 | 0.1973 | 0.2005 |  |
|  | 2.7961 | 0.2026 | 0.2271 | 0.2037 |  |
|  | 2.7044 | 0.2061 | 0.2314 | 0.2074 |  |
|  | 2.6212 | 0.2077 | 0.2392 | 0.2096 |  |
|  | 2.5452 | 0.2198 | 0.2449 | 0.2212 |  |
|  | 2.4754 | 0.2258 | 0.3009 | 0.2302 |  |
|  | 2.4111 | 0.2467 | 0.2605 | 0.2473 |  |
|  | **Overall** | 0.2023 | 0.2346 | 0.2039 |  |

# Rxpct factors in resolution bins at cycle 3


|  |  |  |  |  |  |  |  |  |
| --- | --- | --- | --- | --- | --- | --- | --- | --- |
|  | **<d> (Å)**  Rxpct\_Work | Rxpct\_Free | Rxpct\_All | |  |  |  |  |
|  | 15.0574 | 0.3589 | 0.4936 | 0.3636 |  |
|  | 8.6934 | 0.2295 | 0.2698 | 0.2319 |  |
|  | 6.7339 | 0.2217 | 0.2469 | 0.2231 |  |
|  | 5.6912 | 0.2089 | 0.2805 | 0.2113 |  |
|  | 5.0191 | 0.1748 | 0.2264 | 0.1779 |  |
|  | 4.5400 | 0.1630 | 0.1861 | 0.1642 |  |
|  | 4.1762 | 0.1712 | 0.1952 | 0.1726 |  |
|  | 3.8878 | 0.1783 | 0.2320 | 0.1807 |  |
|  | 3.6520 | 0.1823 | 0.2338 | 0.1848 |  |
|  | 3.4544 | 0.1813 | 0.2278 | 0.1840 |  |
|  | 3.2858 | 0.1898 | 0.2341 | 0.1924 |  |
|  | 3.1397 | 0.1910 | 0.2128 | 0.1920 |  |
|  | 3.0115 | 0.1903 | 0.2143 | 0.1913 |  |
|  | 2.8978 | 0.1995 | 0.2028 | 0.1997 |  |
|  | 2.7961 | 0.1997 | 0.2384 | 0.2015 |  |
|  | 2.7044 | 0.2056 | 0.2310 | 0.2069 |  |
|  | 2.6212 | 0.2055 | 0.2348 | 0.2072 |  |
|  | 2.5452 | 0.2207 | 0.2526 | 0.2225 |  |
|  | 2.4754 | 0.2264 | 0.3039 | 0.2309 |  |
|  | 2.4111 | 0.2495 | 0.2757 | 0.2506 |  |
|  | **Overall** | 0.1986 | 0.2362 | 0.2005 |  |
